# Reply: Methodological Considerations on Targeted Polymer Embolization in Obstructive Hypertrophic Cardiomyopathy

**DOI:** 10.1016/j.jaccas.2026.108068

**Published:** 2026-05-13

**Authors:** Evandro Martins Filho, Thiago Schumann Munhoz, César Henrique Morais Alves, Edvaldo Ferreira Xavier Júnior, Sidney Munhoz Júnior

**Affiliations:** a Santa Casa de Misericórdia de Maceió, Maceió, Alagoas, Brazil; b Instituto Dante Pazzanese de Cardiologia, São Paulo, Brazil; c Laboratório de Hemodinâmica e Cardiologia Intervencionista do Centro-Oeste, Cuiabá, Brazil

**Figure:**
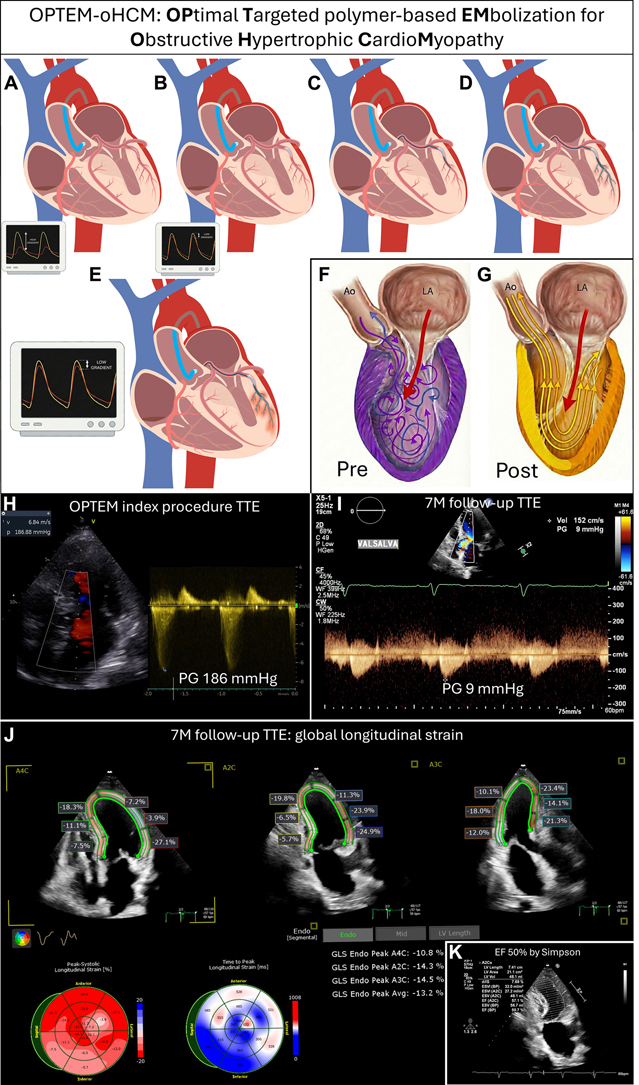

In the OPTEM (OPtimal Targeted Polymer-based EMbolization for Obstructive Hypertrophic CardioMyopathy) approach (Figures 1A to 1E), target vessel selection is primarily physiology-driven.^1^ In our case, balloon occlusion of the obtuse marginal branch under dobutamine stress produced immediate and reproducible hemodynamic resolution (Figures 1A to 1B), establishing a direct causal link between the targeted myocardial territory and the obstructive dynamics. Although myocardial contrast echocardiography is the gold standard for delineating anatomical perfusion territories, it does not inherently predict hemodynamic relevance in atypical phenotypes. In complex oHCM, reliance on anatomical imaging alone may therefore result in functional false negatives, failing to identify vessels that are critical to the obstructive mechanism.

**Figure 1:**
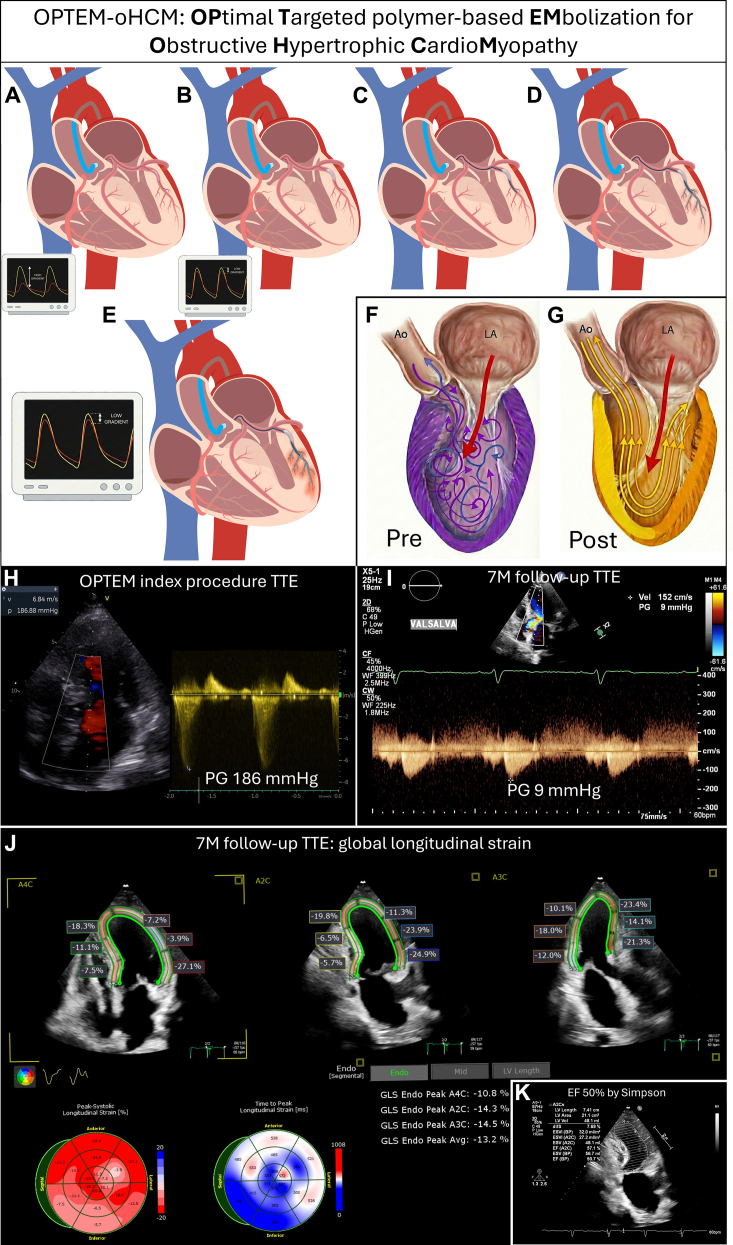
OPTEM-oHCM: Physiology-Guided Polymer Embolization for Obstructive Hypertrophic Cardiomyopathy: Procedural Steps, Mechanistic Concept, and 7-Month Follow-Up (A and B) Balloon occlusion test in the obtuse marginal branch. (A) Hypercompliant balloon positioned in the marginal branch (deflated). (B) Balloon inflation producing immediate reduction of the left ventricular outflow tract (LVOT) gradient, confirming the functional relevance of the targeted territory. (C) Microcatheter advanced parallel to the balloon within the target vessel; the balloon is reinflated to prevent proximal reflux. (D and E) Controlled polymer injection through the microcatheter with the balloon maintained inflated to ensure distal distribution. (F and G) Mechanistic illustration. (F) Baseline obstructive physiology with maladaptive systolic force vectors contributing to LVOT obstruction (Pre). (G) After embolization (Post), realignment of contraction vectors restores laminar systolic flow toward the aorta. (H and I) Transthoracic echocardiography showing severe obstruction at the index procedure (peak gradient 186 mm Hg) and sustained reduction at 7-month follow-up during Valsalva (peak gradient 9 mm Hg). (J and K) Seven-month strain imaging demonstrating left ventricular ejection fraction of 50% and global longitudinal strain of −13.2%, consistent with durable physiological remodeling.

Left ventricular outflow tract obstruction is increasingly recognized as multifactorial. Hermida et al^2^ demonstrated that obstructive phenotypes involve complex global remodeling beyond basal septal thickness. Similarly, Sawma et al^3^ showed that severe obstruction can occur independent of massive hypertrophy, driven instead by acute septal-to-outflow angulation. This underscores obstruction as a vector-dependent phenomenon. Mechanistically, the obtuse marginal territory likely contributed to adverse systolic force vectors driving the intraventricular gradient. The resulting gradient reduction is best explained by a geometry-mediated realignment of contraction vectors after embolization, altering intraventricular flow dynamics, rather than global depression of myocardial contractility, a mechanism further supported by the durability of the hemodynamic effect (Figures 1F to 1G).

Regarding the distinction between mid-ventricular and outflow gradients, Dr Kivrak and colleagues suggest that lateral wall dysfunction might preferentially attenuate mid-ventricular obstruction. However, our invasive hemodynamics demonstrated complete equalization of left ventricular and aortic pressures. Had the intervention merely suppressed a mid-ventricular component while leaving the basal obstruction intact, a residual subaortic gradient would have persisted.

Finally, the durability of the result argues against a transient myocardial stunning mechanism. At 7-month follow-up, the patient remained clinically stable in NYHA functional class I. Strain-enhanced transthoracic echocardiography confirmed persistent abolition of the intraventricular gradient, with a residual peak of 9 mm Hg even during the Valsalva maneuver, alongside a mildly reduced left ventricular ejection fraction of 50% and global longitudinal strain of −13.2%, with expected segmental abnormalities (Figures 1H to 1K). This reduction in ejection fraction reflects resolution of the pathological hyperdynamic state characteristic of obstructive hypertrophic cardiomyopathy rather than pump failure, representing a deliberate and clinically justified trade-off in a high-risk patient without surgical options.

In conclusion, while myocardial contrast echocardiography remains the gold standard for anatomical delineation, OPTEM-oHCM serves as a complementary, physiology-guided strategy for selected patients in whom anatomical dogmas fail to address the hemodynamic reality.

## References

[1] Martins Filho E., Munhoz T.S., Alves C.H.M. (2025). OPTEM-oHCM: optimal targeted polymer-based EMbolization for obstructive hypertrophic cardiomyopathy. JACC Case Rep.

[2] Hermida U., Stojanovski D., Raman B. (2023). Left ventricular anatomy in obstructive hypertrophic cardiomyopathy: beyond basal septal hypertrophy. Eur Heart J Cardiovasc Imaging.

[3] Sawma T., Schaff H.V., Karadzha A. (2025). Outcomes of septal myectomy in patients with obstructive hypertrophic cardiomyopathy and minimal septal hypertrophy. J Thorac Cardiovasc Surg.

